# Supplementary material for: Venomix: a simple bioinformatic pipeline for identifying and characterizing toxin gene candidates from transcriptomic data
Source: PeerJ. 2018 Jul 31;6:e5361. doi: 10.7717/peerj.5361 (PMC6074769; doi:10.7717/peerj.5361)
Supplement: Supplemental Information 3 [file peerj-06-5361-s003.gz › FinalOutput_E-20/Snake_venom_vascular_endothelial_growth_factor_toxin_VR-1_1/finaltree.pdf]

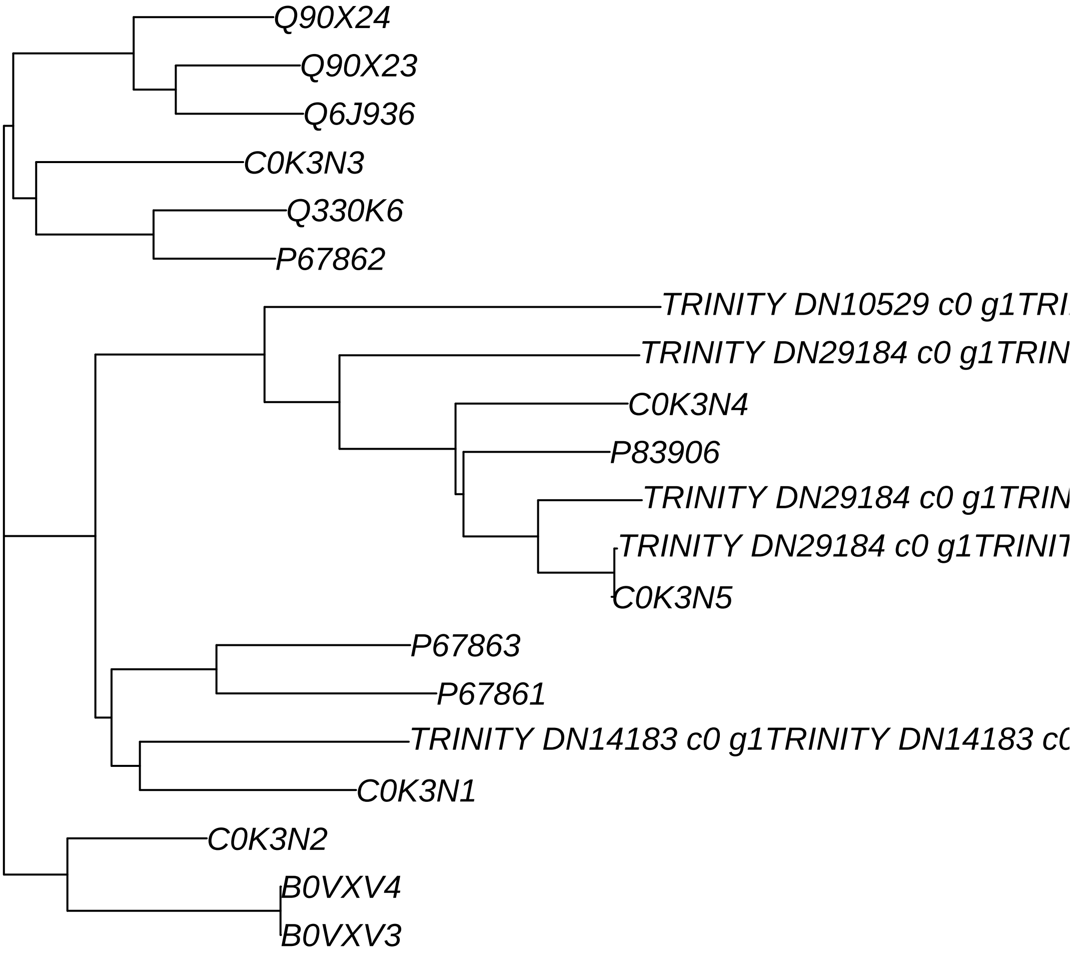
Q90X24
Q90X23
Q6J936
C0K3N3
Q330K6
P67862
TRINITY DN10529 c0 g1TRI
TRINITY DN29184 c0 g1TRIN
C0K3N4
P83906
TRINITY DN29184 c0 g1TRIN
TRINITY DN29184 c0 g1TRINIT
C0K3N5
P67863
P67861
TRINITY DN14183 c0 g1TRINITY DN14183 c
C0K3N1
C0K3N2
B0VXV4
B0VXV3
